# Expertise in Allergy and Immunology Can Aid Other Medical Specialties

**DOI:** 10.1097/WOX.0b013e3181b71c28

**Published:** 2009-09-15

**Authors:** S Gunnar O Johansson

**Affiliations:** 1 Karolinska University Hospital, Stockholm, Sweden

## 

Allergy has come a long way as a medical science since the discovery of Immunoglobulin E (IgE) some 40 years ago. We have become much more proficient in diagnosing and managing classic allergic conditions like asthma, rhinitis, and eczema. However, I am also convinced that allergists and immunologists can make valuable contributions to other medical specialties.

A case in point is anesthesiology. Anaphylaxis during general anesthesia is recognized as a severe problem and a threat to patient safety. Neuromuscular blocking agents (NMBAs) represent the most frequent cause, with suxamethonium (SUX) as the substance most often involved. Other agents are latex, dextran, and various drugs [1].

Together with my friend and colleague Erik Florvaag at Haukeland University Hospital in Bergen, Norway, and my coworkers at the Department of Medicine, Clinical Immunology and Allergy at the Karolinska Institute in Stockholm, I have recently had the satisfaction of bringing new light on this issue. Our findings have had considerable clinical and health economic effects. Through prevention, a sharp decline in IgE sensitization to NMBAs and also cases of anaphylaxis during general anesthesia is seen and is presently under investigation in Norway, a former high-prevalence country.

The starting point for our investigations was an intriguing discrepancy found in the frequency of such reactions in Norway and Sweden, 2 countries geographically and socially closely related. Clinical reports showed that anaphylactic reactions to NMBAs were on the order of 10 times higher in Norway than in Sweden. Because the patient must already be IgE-sensitized to react upon exposure to the NMBA, could there be an unknown, environmental exposure factor?

To look for such a factor, we undertook an environmental exposure study, involving 84 household and other environmental chemicals--skin care ointments, hair care products, cough syrups, lozenges, toothpastes, cleansers, and motor oils--which were collected from the homes of individuals in Bergen and Stockholm, persons that had proven to be sensitized to SUX and nonsensitized persons. We could find no differences in exposure between persons from Sweden and those from Norway, with one crucial exception: In Norway, but not in Sweden, a cough mixture, Tuxi, (Weifa AS, Oslo, Norway), was used that contained pholcodine (PHO), which in principle is a modified morphine (MOR).

To explore the possible effects of this difference in exposure, we decided to perform a comparative study to document the prevalence of IgE sensitization to PHO in the 2 countries. As morphine has been suggested as being suitable for the screening of individuals sensitized to the quaternary ammonium ion epitope, the most likely allergenic epitope for the specific binding of IgE to the NMBA, we also tested for sensitization to MOR. The results showed that in Norway 0.4% of persons who donated blood, 3.7% of patients with allergies, and 38.5% of patients with an anaphylactic reaction to NMBAs were sensitized to SUX. The corresponding percentages for MOR were 5%, 10%, and 66.7%, respectively. Among those from Stockholm who donated blood and patients with allergies, none with antibodies to SUX or MOR were found. IgE antibodies to PHO were found in 6% of persons who donated blood that were from Bergen but in no one from Stockholm [2].

Thrilled by this discovery, we initiated clinical studies to compare the effects on IgE production of Tuxi and PHO-free drugs. A pilot study with individuals IgE-sensitized and nonsensitized showed that PHO exposure caused an extreme, most unexpected, close to 100-fold increase of serum levels of IgE and IgE antibodies to PHO, MOR, and SUX [3]. However, only the IgE-sensitized individuals responded. The origin of the IgE was polyclonal and resembled the IgE response in the graft versus host reaction after bone marrow transplantation [4]. These findings were confirmed in a controlled, randomized clinical trial on a population with previously diagnosed IgE-mediated anaphylaxis toward NMBAs [5].

As a result of these studies, the Norwegian producer decided not to renew the marketing license for Tuxi, which was taken off the market in March 2007. The long-term immunologic and clinical consequences will take some time to document, but just a year after the withdrawal, the percentage of sera that was sent to the Haukeland University Hospital allergy laboratory and found to have IgE antibodies to PHO, MOR, and SUX was just about half as much as when Tuxi was on the market. And even more satisfying, the number of cases of anaphylaxis in connection with general anesthesia in Norway has now, 2 years later, markedly decreased.

The Norwegian findings have also prompted studies in other countries. The fact is that reports of NMBA-induced anaphylaxis are considerably more common in countries such as France and the United Kingdom, which are high consumers of PHO, than in low- or non-consuming countries such as Finland, Germany, and the United States. An international study on the connection between PHO consumption and IgE sensitization in 9 different countries supports the findings in Norway: a high prevalence of IgE sensitization to PHO in high-consumption countries [6]. An extension of this study to southeast Asia is currently in progress where there are many countries, for example, Australia and New Zealand, known to have problems with NMBA anaphylaxis usually combined with high sales of PHO. Withdrawal of PHO-containing drugs seems most urgent in these high-consuming countries.

It has recently come to my attention that a PHO-containing drug was also marketed in Sweden back in the 1970s and 1980s. The cough syrup Tussokon (Pharmacia AB, Uppsala, Sweden) was phased out of the market between 1987 and 1989. To further test the pholcodine hypothesis, we analyzed some Swedish sera from the 1970s and 1980s found in our freezers. As many as 5% to 6% of these sera had antibodies to PHO, whereas only 2% of the sera from the 1990s and none from 2002 or later tested positive for these antibodies [7].

The results of our pholcodine work lend strong support to the opinion that allergists need to be involved in the investigation of cases of anaphylaxis in connection with anesthesia. Cross-disciplinary collaboration can obviously yield results of great clinical and health economic value.

For me, personally, it feels very satisfying to help avoid concern and suffering among patients undergoing surgery and to facilitate the work of surgeons and anesthesiologists.
